# Cancer resistance to immunotherapy: What is the role of cancer stem cells?

**DOI:** 10.20517/cdr.2022.19

**Published:** 2022-10-27

**Authors:** Gourab Gupta, George Merhej, Shakthika Saravanan, Hexin Chen

**Affiliations:** Department of Biological Science, University of South Carolina, Columbia, SC 29208, USA.

**Keywords:** Immunotherapy, cancer stem cells, immunotherapy resistance, tumor microenvironment

## Abstract

Immunotherapy is an emerging form of cancer therapy that is associated with promising outcomes. However, most cancer patients either do not respond to immunotherapy or develop resistance to treatment. The resistance to immunotherapy is poorly understood compared to chemotherapy and radiotherapy. Since immunotherapy targets cells within the tumor microenvironment, understanding the behavior and interactions of different cells within that environment is essential to adequately understand both therapy options and therapy resistance. This review focuses on reviewing and analyzing the special features of cancer stem cells (CSCs), which we believe may contribute to cancer resistance to immunotherapy. The mechanisms are classified into three main categories: mechanisms related to surface markers which are differentially expressed on CSCs and help CSCs escape from immune surveillance and immune cells killing; mechanisms related to CSC-released cytokines which can recruit immune cells and tame hostile immune responses; and mechanisms related to CSC metabolites which modulate the activities of infiltrated immune cells in the tumor microenvironment. This review also discusses progress made in targeting CSCs with immunotherapy and the prospect of developing novel cancer therapies.

## INTRODUCTION

Cancer heterogeneity at the cellular level has recently shifted cancer research from exclusive investigations of events affecting specific cell types to investigations of events governing all types of cells in the tumor microenvironment^[1,2]^. Tumor heterogeneity is the concept of morphological and phenotypical variation in cancer cells and, therefore, different proliferation potential. Cancer stem cell theory is a confirmed theory derived from this topic, which characterizes subpopulations of tumor cells that can proliferate extensively^[3]^. These cells are comparable to normal adult stem cells in that they both undergo self-renewal to maintain the stem cell population. The purpose of this feature is to enable the continuous replacement of cells needed in tissues due to everyday damage, age, *etc*. In cancer stem cells, however, self-renewal has been observed to be the driving force for tumorigenicity and metastasis. Furthermore, there is evidence that cancer stem cell population growth is promoted by oncogenic events induced by chemotherapy, radiation therapy, hypoxia, *etc*., which are generally thought to reduce tumor growth but, on the contrary, increase the risk of cancer recurrence. Cancer stem cells are observed in all cancers^[3]^. While cancer stem cells were shown to have the ability to differentiate into distinct types of cancer cells within the tumor^[4-6]^, several types of immune cells were shown to control cancer growth in different ways, from directly targeting and killing cancer cells to suppressing the immune attack by modulating other immune cells^[7]^. While this paradoxical effect of immune cells on cancer growth is critical to developing adequate immunotherapy regimens, the presence of cancer stem cells in the tumor microenvironment is another factor that could affect the response of cancer to immunotherapy and subsequently lead to therapy resistance. This review discusses the distinctive characteristics of cancer stem cells that we believe may contribute to immunotherapy resistance. Along the same lines, we suggest new areas of focus in the field of immunotherapy research, which may help bypass the therapy resistance caused by cancer stem cells.

## CANCER STEM CELLS

The presence of stem cells in tumors has shifted laboratory and clinical approaches to cancer research and has given insight into mechanisms contributing to the therapeutic resistance of many cancers^[8-12]^. Cancer stem cells (CSCs) are the “seed” of the tumor with self-renewal and differentiation properties. They are believed to be present in all cancers. Although there is debate regarding CSC generation, hypotheses include evolving from the de-differentiation of differentiated cells, the division of existent tissue stem cells, and a combination of both rationales^[13]^. More importantly, CSCs have been observed to resist chemotherapy by at least five main mechanisms: (i) being in a quiescent proliferative state rendering the cells less sensitive to any form of therapy; (ii) activating drug efflux mechanisms; (iii) overexpressing DNA repair mechanisms; (iv) overexpressing anti-apoptotic genes; and (v) releasing cytokines and chemokines rendering other tumor cells resistant to therapy^[14]^. As the mechanisms by which CSCs affect the development of chemotherapy resistance have been extensively reviewed, we focus on the characteristics of CSCs that may contribute to resistance to immunotherapy and consider recent findings related to cancer immunotherapy and the cancer microenvironment.

## THE INTERACTION BETWEEN IMMUNE CELLS AND CANCER STEM CELLS

Current research aims to understand the immune system’s role in cancer as it is evident that the immune system does not target cancer cells in the same way that it targets other types of self-altered cells. Cancer can escape from immunosurveillance through a well-described procedure termed “cancer immunoediting”, which comprises three main sequential events, namely elimination, equilibrium, and escape, eventually leading to cancer growth^[15]^. A tumor microenvironment is an ideal place for starting an investigation of factors contributing to therapy resistance because it is a diverse environment in which a variety of cells grow and interact with one another. It has been shown that the tumor microenvironment (TME) is rich with factors that suppress immune cells by inhibiting their growth.

This interaction in TME can involve both innate and adaptive immune systems. Innate immune cells integral for tumor recognition include natural killer (NK) cells, dendritic cells (DC), macrophages, and myeloid-derived suppressor cells (MDSC). Adaptive immune cells include T cells and B cells. The unique properties of CSCs may make them more susceptible or resistant to immune cells. For example, CSCs have been observed to be more susceptible to being killed by NK cells because of their typical presentation of no/low levels of MHC class I^[16]^. Additionally, CSC-secreted factors can actively modulate the function of immune cells to generate an immune-suppressive environment. Tumor-associated macrophages (TAMs) can be differentiated into an M1 or M2 phenotype via exposure to specific cytokines and metabolites in the TME. M1 macrophages are characterized by phagocytic activity promoting tumor-killing properties, whereas M2 macrophages are characterized by cytokine production promoting an anti-inflammatory response in the TME, thus promoting tumor growth. CSCs have been observed to release many cytokines, chemokines, and soluble proteins to attract macrophages into the TME and polarize them into M2 TAMs^[17]^. Furthermore, CSC-induced secretion of the immunosuppressive cytokine IL-10 increased the differentiation of CD4^+^CD25^+^FoxP3^+^ regulatory T cells^[18]^. Regulatory T cells are a type of CD4^+^ T cell that function as an immunosuppressant to prevent inappropriate immune system activation. These immunosuppressive cells can interact with cancer cells in various ways ranging from direct ligand-receptor interactions to systemic regulation^[19,20]^. While systemic regulation is beyond the scope of our discussion in this review paper, it is essential to mention that altered hematopoiesis in cancer is a growing field of research^[21,22] ^that may contribute to immunotherapy resistance.

### CSCs and resistance to immunotherapy

The mechanisms that govern the resistance of cancer to any form of therapy are complicated and numerous. No single mechanism has been shown to fully govern the resistance of a tumor to shrinkage in response to therapy. Instead, a combination of alterations related to surface receptors, secreted factors, and metabolism have been noted in therapy-resistant cells^[23]^. Therefore, understanding the behavior of all cells in the TME is crucial to fully understanding therapy resistance. As mentioned above, CSCs can contribute to chemotherapy resistance by either modifying their own behavior or by inducing a therapy-resistant phenotype in surrounding cancer cells. It is also important to mention that the mechanisms governing resistance to immunotherapy may differ from those governing resistance to chemotherapy. 

Cancer immunotherapy is briefly divided into two main types of interventions: passive and active^[24]^. Passive immunotherapy strategies aim to compensate for missing or deficient immune functions by directly administering tumor-specific antibodies, recombinant cytokines, or the adoptive transfer of immune cells. Active immunotherapy strategies are designed to stimulate effector functions *in vivo*, e.g., vaccination strategies with tumor peptides^[25]^ or allogeneic whole cells^[26]^, inhibition of immune checkpoints with antibodies, and induction of immune responses with oncolytic viruses. Unlike chemotherapeutic drugs, which directly target cancer cells, the success of immunotherapy relies on the dynamic interaction between cancer and immune cells. Increasing evidence indicates that non-genetic, intrinsic cancer cell alterations play a key role in resistance to immunotherapies and immune evasion. Therefore, understanding therapy resistance in particular requires understanding the interactions among immune cells, cancer cells, and CSCs and the behavior of CSCs in response to immunotherapy, as CSCs are considered the root of cancer.

CSCs were first identified from leukemia cells as a subpopulation of cancer cells that displayed increased tumorigenicity using serial limiting dilution transplantation assays with severe combined immunodeficient (SCID) mice^[27]^. Thereafter, CSCs in many solid tumors were identified using nonobese diabetic (NOD)/SCID mice^[6]^. Most of these experiments, using partially immunocompromised mice, demonstrated that a small subset of cancer cells can initiate tumor development. It is still being debated whether the increased tumorigenicity of CSCs attributes to the “stemness” or adaptation ability of CSCs to new microenvironments^[28,29]^. Indeed, it has been reported that tumor-initiating cells in human melanoma were not a rare population when transplanted into more highly immunocompromised NOD/SCID IL-2 receptor gamma chain null (NSG) mice^[30]^. Xenotransplantation of non-CSCs into NSG mice also resulted in tumor formation. However, in tumor tissues developed from non-stem melanoma cells transplanted into NSG mice, the expressions of melanoma markers were no longer retained, suggesting the failure of self-renewal. Moreover, these non-stem cancer cells also failed to pass in NSG mice, whereas CSCs were successively propagated and, more importantly, mirrored the heterogeneity of the parental melanoma^[29]^. Similar findings have been reported in other models^[31]^. The current view is that the rarity of cancer cells exhibiting tumor-initiating properties may not be so important for CSC definition^[32]^. Instead, tumor immune evasion may be a more important characteristic of CSCs. Consistently, tumor tissues exposed to an immune challenge *in vivo* exhibited a CSC-like gene expression profile with increased CSC properties, including tumor initiation ability^[33]^. Cancer stem cells have characteristics such as slow rates of division, heightened activation of DNA repair mechanisms, cellular plasticity, and microenvironment characteristics including hypoxia and acidosis, which are due to the expression of specific surface markers and secreted and/or enzymatic proteins. Many of these factors may also contribute to the resistance of CSCs to immunotherapy. These mechanisms can be organized into three main categories: (i) mechanisms related to cellular surface proteins; (ii) mechanisms related to released cytokines; and (iii) mechanisms related to metabolic alteration.

### Mechanisms related to cellular surface proteins

CSCs are known to express a wide variety of cellular markers that can be used to differentiate them from other tumor cells^[34,35]^. In addition to “stemness” markers, CSCs also express membrane proteins that play roles in tumorigenesis and/or therapy resistance^[36]^. This review discusses the markers that are vital in the context of cancer immunology and resistance to immunotherapy.

#### Immune recognition molecules

One of the mechanisms used by CSCs to evade immune response is the downregulation of major histocompatibility complex class I (MHC I) molecules. This protects them from recognition by CD8^+^ T cells. For example, enriched cells with stem-related markers isolated from patients with locally advanced head and neck squamous cell carcinoma (HNSCC) showed decreased expression of human leucocyte antigen I (HLA-I) molecule after chemotherapy treatment^[37]^. Similarly, CSCs isolated from primary melanoma, glioblastoma multiforme (GBM) and lung cancer cell lines displayed lower MHC-I levels than their differentiated counterparts^[18,38-40]^. Downregulating MHC-I may make CSCs more susceptible to being killed by NK cells. However, CSCs from acute myeloid leukemia (AML)^[41]^, GBM^[42]^, and breast cancer (BC)^[43]^ were found to escape NK cell-mediated killing by the downregulation of activating NKG2D ligands. Lastly, the upregulation of the “don’t eat me” signal, CD47, is another way for CSCs to evade immune control^[44]^. The blocking of CD47 was shown to enable macrophage-mediated phagocytosis of CSCs from pancreatic ductal adenocarcinoma (PDAC), AML, and hepatocellular carcinoma (HCC), and thus it promotes their elimination^[45-47]^.

#### Immune checkpoint proteins 

PD-L1/PD-1 has been well established as an immune checkpoint to control T cell function. CSCs isolated from GBM, BC, CRC, and HNSCC can evade immune surveillance by increasing the expression of the immune checkpoint ligand PD-L1^[48]^, which binds to its receptor, PD-1, expressed on T-cell surfaces, thus inducing their exhaustion^[48]^. Another immune “brake” is represented by T cell immunoglobulin mucin-3 (TIM-3), a specific surface molecule found on leukemic stem cells^[49]^. This receptor was described as responsible for T-cell suppression and MDSC expansion. Similarly, it was observed that B7-H4 promotes brain CSC tumorigenicity while negatively regulating T cell-mediated immunity^[50]^.

#### Immuno-stimulatory molecules 

CD80 is a co-stimulatory molecule known for its role in T-cell activation. It is primarily expressed by immune cells but has been shown to be expressed in cancer cells including melanoma, colorectal cancer, and some tumor-initiating stem cells^[51,52]^. CD80 is an important cellular surface protein that has been shown to contribute to stem cell resistance to immunotherapy^[52]^. In one study conducted on a model of epidermal squamous cell carcinoma, CD80 in CSCs was shown to be indispensable for CSC resistance to adoptive cytotoxic T cell transfer therapy (ACT)^[52]^. Interestingly, CD80 was also observed to interact with cytotoxic T lymphocyte antigen-4 (CTLA-4), which is known to suppress T cell function^[53]^. Upon engaging CTLA-4, CD80-expressing CSCs directly dampen cytotoxic T-cell activity, which provides evidence for the importance of CD80 in immunotherapy resistance. The mechanism of action, however, needs to be confirmed by future research. In the same study, the authors selected CSCs that displayed sensitivity to TGFβ and studied the resistance of those CSCs to adoptive cell transfer (ACT) therapy. TGFβ responding CSCs were shown to be resistant to ACT, and, interestingly, CD80 expression was observed to be indispensable for resistance. However, interactions between these two proteins have not been studied^[52]^. Future research is required to understand the possible overlap in the mechanisms governing TGFβ sensitivity and CD80 in these resistant cells. Moreover, the clinical feasibility of specifically targeting CSCs’ CD80 while sparing CD80 expressed by other immune cells is also not well understood. In a more recent study, both *in vitro* and *in vivo* deactivation of CD80 were associated with higher immunogenicity manifested by the activation of immune cells^[54]^. This immunogenicity was antagonized by blocking CTLA-4^[54]^. Although the aforementioned study does not specifically target CSCs, it confirms two important concepts governing the mode of action of CD80 (shown in the previously mentioned study to be highly expressed by TGFβ sensitive CSCs): (i) CD80 has an anti-immunogenic role in at least some types of cancer; and (ii) CD80 exerts its anti-immunogenic functions, at least in part, through CTLA-4. 

Apart from the above-mentioned mechanisms, CSCs further interact with immune cells in order to create a suitable microenvironment for its survival and self-renewal. Studies have shown the importance of TAMs in maintaining tumor homeostasis and immune suppression. One such interaction involves the myeloid cell marker CD11b on TAMs and CD90 on cancer stem cells, accompanied by EphA4 and epinephrine leading to the release of several other immunosuppressive cytokines that help maintain cancer stemness and survival^[55]^. CSCs can also interact with CD66^+^ neutrophils to prevent their maturation into N2 neutrophils alongside blocking T cell IFNγ-mediated killing of cancer cells^[56,57]^. Another study showed the importance of CSCs surface marker CD133, which prevents DC maturation and thus downregulates T cell-mediated response^[58]^.

### Mechanisms related to released cytokines

The release of cytokines has been observed to have paradoxical effects on immunotherapy resistance. While proinflammatory cytokines are required for adequate function of T cells in ACT and in CTLA-4/PD-1 targeting therapies, they can induce stemness of CSCs, promoting therapy resistance^[36]^. Many cytokines have dual roles in promotion of CSC self-renewal and immune evasion and immune suppression. In this context, two main therapy options arise: (i) moderating the proinflammatory environment in a way that can induce/maintain stemness; and (ii) antagonizing the proinflammatory environment in a way that can block stemness, at least partially, yet create an immunosuppressive environment that may not be favorable for immunotherapy.

Tumor microenvironments in solid tumors are rich in many cytokines, including IL-1, IL-6, IL-4, IL-8, granulocyte-CSF, MF inhibitory cytokine-1 (MIC-1), and TGF-β, which can impair the anti-tumor immune responses and shield them from T-cell infiltration^[59]^. IL-1β is responsible for the infiltration of myeloid cells, primarily MDSCs, CD11b^+^Gr-1^+^ granulocytes, and CD11b^+^F4/80^+^Gr-1^−/low^ TAMs in the tumor microenvironment, thereby promoting tumor growth, progression, and poor prognosis^[60,61]^. IL-6 produced in the tumor microenvironment by CSCs has been shown to enhance the neoplastic progression of tumors. STAT3 activation by IL-6 in TAMs and MDSCs induces the expression of VEGF and bFGF in a positive feedback mechanism promoting angiogenesis^[62]^. Apart from regulating the TAMs and MDSCs, IL-6 has also been shown to interfere with the antigen presentation of DCs that are primarily important for the activation and priming of CD8^+^ T cells (cytotoxic T cells)^[62]^. Furthermore, IL-6 signaling also induces the polarization of immature myeloid cells to macrophages or MDSCs instead of antigen-presenting DCs, which in turn contributes to tumor progression. IL-8, for example, has been shown to recruit and activate the differentiation of immature neutrophils into pro-tumor neutrophils in the tumor microenvironment, thereby helping tumor progression and angiogenesis^[63]^. These pro-tumor neutrophils can suppress the adaptive immune response by secreting arginase 1, which cleaves the arginase required by the cytotoxic T cells to function^[64,65]^. CSCs were shown to promote the polarization of macrophages toward an M2 phenotype by the production of TGF-β, granulocyte-macrophage colony-stimulating factor (GM-CSF), and macrophage colony-stimulating factor (M-CSF)^[66]^ and via the cyclooxygenase (COX)-2/PGE2 pathway^[67]^. In turn, M2 TAMs support the expansion and drug resistance of CSCs by producing the cytokine IL-6^[68]^. Administration of TGF-β inhibitors eliminates TGF-mediated immunosuppressive effects on the immune system, thereby enhancing immunotherapy efficiency^[69]^.

Apart from their role in immune suppression, many cytokines, including TNFα^[70]^, IL-17^[71]^, IL-1^[72]^, and IL-8^[64]^, are known to activate CSC formation and renewal. For example, a recent study showed that IL-17 could activate the autophagy of CSCs through one of its receptors, IL-17RB, in a gastric cancer model^[73,74]^. While autophagy is a cell death mechanism, it may be indispensable for the self-renewal of CSCs and can be considered as a homeostatic mechanism through which the tumor’s pool of CSC is replenished.

### Mechanisms related to metabolic alteration

Although poorly studied, metabolic alterations in CSCs might be vital for the adequate understanding of many aspects of the tumor microenvironment^[75,76]^. Metabolic alterations in CSCs have been shown to govern cancer-related mechanisms that may serve purposes beyond energy production^[76]^. In other words, metabolic alterations might go hand in hand with alterations in both stemness and growth potential. For example, the overproduction of lactate has always been thought of as a demonstration of the tumor’s adaptation to hypoxia and is now known to have roles in the self-renewal of CSCs. We focus our discussion on CSC metabolic plasticity, which may govern resistance to immunotherapy and be a potential therapy target^[77]^.

### Excessive lactate production

Although studies have shown that CSCs can undergo oxidative phosphorylation, the glycolysis/lactate production rate has been shown to be significantly higher in CSCs than in non-CSCs of a tumor^[78]^. Moreover, CSCs were also shown to be activated by lactate^[79]^. One recent study showed that lactate, produced by differentiated cancer cells from a colorectal cancer patient, was shown to promote the self-renewal of CSCs in organoids^[80]^. Therefore, lactate represents an important metabolite that is secreted by CSCs and responded to by CSCs. Lactate can induce the accumulation of MDSCs through granulocyte-macrophage colony-stimulating factor (GM-CSF) and IL-6 and enhance the immunosuppressive phenotype of MDSC through the G-protein-coupled receptor 81 (GPR81)/mTOR/HIF-1a/STAT3 pathway^[81,82]^. In addition, lactate-induced HIF-1a activates the CCL20/CCR6 axis by inducing myeloid trigger receptor-1 (TREM-1) expression in TAMs, attracting aggregation and initiating immunosuppressive effects of Treg. A recent study showed that lactate-derived lactylation of histone lysine residues serves as a new epigenetic modification that directly stimulates gene transcription from chromatin^[83]^. Histone lysine lactylation has been shown to promote M2 activation, but the exact mechanism is not well understood^[83]^. Besides, lactate inhibits the expression of activated receptor NKp46 in NK cells^[81]^. What is yet to be determined, however, is whether targeting lactate metabolism would affect the response of tumor cells to immunotherapy by antagonizing the stemness in CSCs.

Lactic acid accumulation in the tumor microenvironment is a well-established immunosuppressive strategy employed by CSCs. Studies have shown that the increased accumulation and production of lactate can hinder tumor infiltration of NK cells and T cells^[84]^ as well as inhibit proliferation and the production of cytokines by T cells^[85]^. A very recent article also discusses the role of lactate in the tumor microenvironment regulating T-cell redox functions and suppressing T-cell proliferation, wherein the authors showed that inhibiting lactate production by cancer cells can improve T-cell functions in adaptive T-cell therapy and overcome immunotherapeutic resistance^[86]^. The acidic microenvironment also impairs IL-2 and IFNγ production by T cells, thus reducing the cytotoxic effects of effector T cells^[87]^.

### Retinoic acid metabolism in CSCs

ALDH1 is a putative CSC marker in a variety of solid cancers including pancreatic cancer, ovarian cancer, uterine cancer, and breast cancer^[88]^. Several isoforms (ALDH1A1, ALDH1A2, and ALDH1A3) play a role in retinoic acid (RA) formation through oxidation of all-trans-retinal and 9-cis-retinal that are involved in retinoid signaling, which has been related to the stemness of CSCs. Although it has been observed that RA has anti-tumor and anti-CSC capabilities, there is evidence that its role is manipulated by TME and associated with the ALDH1A3 receptor to promote CSC stemness^[89]^. Sullivan *et al.* observed that RA metabolism via the ALDH1A3 receptor transcriptionally upregulated tissue transglutaminase in mesenchymal glioma CSCs, an enzyme associated with aggressive tumors and upregulation of CD44^+^ glioma CSCs^[90]^. They further noted that glioma CSCs regulated this mechanism by promoting the transcription of RA-inducible genes. Marcato *et al.* found a similar correlation regarding the promotion of RA-induced genes in aggressive and/or triple-negative breast cancer cell lines upon ALDH1A3 and RA interaction^[91]^. 

This interaction may be a potential factor in immunotherapeutic resistance. Terzuoli *et al.* found that ALDH3A1 overexpression in both melanoma and non-small-cell lung carcinoma transcriptionally modified the cells to CSC phenotype with upregulation of PDL-1 and EMT markers and an increase in proinflammatory immunosuppressive factors including NFκB, prostaglandin E2, IL-6, IL-13, and COX-2^[92]^. Additionally, Devalaraja *et al.* uncovered that TME-induced tumor-derived RA induced by IL-13 in sarcoma cells supported immunosuppression via inhibiting monocyte differentiation of antigen-presenting dendritic cells (DCs) and, instead, promoted differentiation of M2 TAMs^[93]^. It is likely that CSCs contribute to this interaction as studies have observed their involvement in both inhibiting DC maturation and promoting M2 TAM differentiation from monocytes^[94]^.

### Altered metabolic pathways in TAM

Solid tumors are known to have special metabolic characteristics that would allow them to face challenges related to hypoxia and/or shortage of nutrients^[77,95]^. It has been shown that hypoxia in the tumor microenvironment preferentially upregulates OXPHOS and FAO metabolic pathways in the M2 TAMs. This causes the accumulation of metabolic byproducts such as glutamine, a-ketoglutarate, and succinate, resulting in activation of the HIF-1α-mediated cell-self-renewal signaling pathway. Furthermore, tumor-associated macrophages undergo “M2 polarization” by secreting IL-6 to promote PI3K/AKT phosphorylation. This promotes protein kinase 1 (PDK1)-mediated phosphoglycerate kinase 1 (PGK1)-catalyzed glycolysis of macrophages^[96]^. Interestingly, IL-6, as discussed in a previous section, can enhance the stemness of CSCs and CSC-dependent chemoresistance through STAT3 transcription factor activation. Other studies have shown a mutually symbiotic relationship between CSCs and M2 TAMs, in that CSCs were considered to play an active role in M2 polarization, resulting in inhibition of antigen presentation and anti-tumor cytotoxic CD8^+^ T-cell responses^[17]^

### Immunotherapeutic strategies to target CSCs

To achieve complete regression of tumors, CSCs have to be targeted for therapy. Compared to differentiated counterparts, CSCs are known to express a different set of genes that can potentially serve as tumor antigens. Although all over-expressed antigens might not be strong immunotherapeutic targets, certain other types of overexpressed antigens have been studied, such as ALDH1A1 and hTERT in CD44^+^ breast cancer CSCs^[97]^, HER2 proto-oncogene in glioma CSCs^[98]^, and CEP55 and COA-1^[99]^ in colon CSCs. These overexpressed antigens could be novel targets for the development of CSC-associated immunotherapeutic strategies. Dendritic cells (DCs) are antigen-presenting cells in the immune system that induce primary immune system responses. In the creation of DC-based vaccines, DCs are primed with tumor antigens, such as fusion with tumor lysates and transfection of certain peptide sequences to induce T-cell activation upon vaccination^[100]^. Examples of recent vaccines being studied targeting CSCs include DCs primed with CSC lysate, Panc-1 CSC lysate, NANOG peptide, and ALDH^high^ CSCs^[101]^. In a study conducted by Yin *et al.*, DCs were primed with pancreatic CSC lysates identified via culturing Panc-1 cells with a non-adherent sphere culture system due to the heterogeneity of surface markers. Upon co-culturing with lymphocytes, proliferation and significant activation of lymphocytes were observed along with the secretion of tumor-killing cytokines INF-γ and IL-2. Significant cytotoxic effects induced by the primed DCs were also observed^[102]^.

Oncolytic virotherapy utilizes viruses engineered to replicate within tumor cells via the cytolytic pathway. The virus is selected regarding the type of cancer and its known receptors to increase infectivity of the tumor cells^[103]^. Examples of viruses used include the herpes simplex virus, adenovirus, vaccinia virus, and measles virus^[101]^. Sato-Dahlman *et al.* conducted a study designing and testing a form of oncolytic virotherapy for colorectal cancer with an adenovirus. The adenovirus was selected in particular because of its capability for efficient transduction in cells with its receptor to combine with the engineered specificity for the CD133 receptor known to be upregulated in colorectal cancer, among other types of cancer. The transmembrane receptor CD133 has been highly studied as a cancer stem cell marker, and its upregulation has been associated with the maintenance of self-renewal and metastasis. The results support increased infectivity and lysis of CD133^+^ cells *in vitro* and *in vivo*^[104]^.

Some CSC-specific surface markers can be used as specific targets for chimeric antigen receptor T cell (CAR-T) therapy to eliminate CSCs. In addition, the expression of MHC molecules on the surface of CSCs is low, which causes MHC restriction when immunotherapy is used to target CSCs^[39]^. However, in CAR-T therapy, CAR-T cells can recognize the target antigen with no MHC restrictions, which endows some advantages for the application of CAR-T therapy to eliminate CSCs. Recently, the clinical application of CAR-T therapy has made an unprecedented breakthrough in the treatment of hematological diseases^[105]^. The safety and feasibility of CAR-T therapy in the treatment of solid tumors have also been confirmed^[106]^. The discovery of surface markers of CSCs provides specific therapeutic targets for the treatment of CSCs. Many previous experiments have identified the expression of CD133, CD90, ALDH, and EpCAM in CSCs of many types of cancer^[107-109]^. These markers can be used as an important molecular target for CAR-T cells to kill CSCs in order to achieve the therapeutic effect of eliminating CSCs and inhibiting tumor recurrence and metastasis. In addition, certain molecular markers expressed in common tumor cells, such as epidermal growth factor receptor variant III (EGFRvIII), chondroitin sulphate proteoglycan 4 (CSPG4), human epidermal growth factor receptor 2(HER2), NKG2D ligands (NKG2DLs), *etc*., are also expressed on the surface of tumor stem cells^[110]^. The construction of CAR-T cells with molecular markers of CSCs as targets has a certain theoretical effect on the elimination of CSCs.

TIL follows the same methodology, but it isolates T cells with tumor-specific epitopes to target antigens rather than the CAR receptor. Chen *et al.* employed this strategy in their observation of the synergistic effect between γδ T cells and CD8^+^ T cells in killing CSCs. This was achieved because γδ T cells secrete interferon gamma (IFN-γ), resulting in the upregulation of MHC class I and CD54/ICAM-1 in CSCs and, thereby, allowing CSCs to become more susceptible to CD8^+^ T cell-mediated cytotoxicity^[111]^. 

While low expression of MHC-I may limit CD8 T-cell recognition and response to CSCs, the lack of MHC-I molecules should, in turn, promote NK cell activation, representing an alternate immunotherapeutic target^[112]^. In regard to adoptive immunotherapy of NK cells, NK cells have been identified to contain activating and cytotoxic receptors that can bind with tumor-specific stress-induced ligands, thereby increasing antigen recognition^[113]^. There is evidence that NK cells may preferentially target CSCs because of both this and their ability to identify the lower expression of MHC class I molecules that provide CSCs their non-proliferative properties. This was supported in the study conducted by Ames *et al.* in their identification of CSC markers CD24^+^/CD44^+^, CD133^+^, and aldehyde dehydrogenase bright present on NK cells *in vitro* and *ex vivo*^[114]^. CSC ligands MICA/B, Fas, and DR5, involved in NK activation, were also noted to be upregulated. The mechanism for this observation was further investigated by studying the NKG2D pathway that is important for the binding of MHC class proteins during cellular stress for increased cytotoxicity. Significant *in vivo* studies have found reduced CSC population and tumor size in NSG mice with human pancreatic tumors^[114]^.

## CONCLUSION AND FUTURE PERSPECTIVES

**Figure 1:**
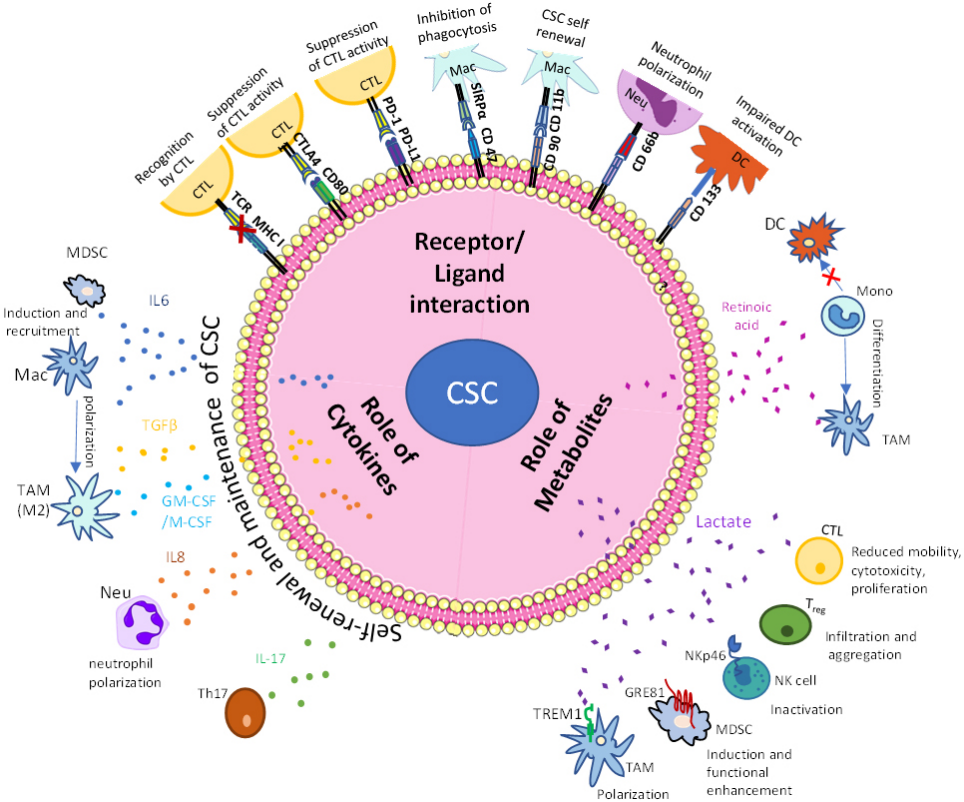
CSC-mediated immune escape and self-renewal. The tumor microenvironment is home to a lot of immune cells, which contribute to cancer cell survival and proliferation. The CSCs can self-renew themselves due to their stem-like properties and are also aided by different cytokines in the TME. Metabolic products from the CSCs, such as retinoic acid and lactate, are also involved in their self-renewal. Other immune cells in the TME, such as MDSCs, immature neutrophils, and TAMs, are also involved in tumor proliferation by dampening the cytotoxic effects of killer T cells and promoting regulatory T cells. CSCs: Cancer stem cells; TME: tumor microenvironment; TAMs: tumor-associated macrophages; CTL: cytotoxic T lymphocyte; Mac: macrophage; Mono: monocyte; Neu: neutrophil; DC: dendritic cells; Treg: regulatory T cell.

Many studies performed in the past decade confirm the importance of the whole microenvironment in driving cancer growth and resistance to therapy. As immunotherapy targets a very fundamental aspect of the tumor microenvironment, namely the interaction between immune cells and cancer cells, investigating the tumor microenvironment is critical for an adequate understanding of therapy options and therapy resistance. This review focuses on CSC characteristics that we think might govern the resistance of cancer to immunotherapy [Figure 1]. While the focus is on the expression of cellular markers/release and response to cytokines/metabolic reprogramming, other mechanisms might govern CSCs’ ability to bypass or help cancer cells bypass immunotherapy. In addition to answering the questions raised in this review paper, future research needs to focus on other aspects that might govern resistance. These include the systemic control of the hematopoiesis characteristic of cancer and the common markers between CSCs and embryonic stem cells, which might allow for escaping immune surveillance and, therefore, escaping immunotherapy.

## DECLARATIONS

### Copyright

© The Author(s) 2022.
